# Social Support Interventions and Post‐Trauma Psychopathology in High‐Risk Professionals: A Scoping Review

**DOI:** 10.1002/cpp.70274

**Published:** 2026-04-15

**Authors:** Maria Eșanu, Elisa van Ee, Vera Prins, Mailer Al‐Rabadi, Luka Potgieter, Miriam Lommen

**Affiliations:** ^1^ Department of Developmental Psychology University of Groningen Groningen the Netherlands; ^2^ Radboud University Behavioural Science Institute Nijmegen the Netherlands; ^3^ Psychotraumacentrum Zuid Nederland, Reinier van Arkel, Den Bosch Den Bosch the Netherlands; ^4^ Department of Clinical Psychology and Experimental Psychopathology University of Groningen Groningen the Netherlands; ^5^ Department Trauma Centre, Beilen GGZ Drenthe Mental Health Institute Beilen the Netherlands

**Keywords:** post‐trauma psychopathology, prevention, social support, treatment, workplace trauma

## Abstract

High‐risk professionals are often exposed to traumatic events at work. Social support is a key protective factor for mental health outcomes, making it a potentially effective component for interventions targeting high‐risk professionals. This review aimed to identify interventions that incorporate social support and highlight the aspects that influence their effectiveness for the prevention and treatment of post‐trauma psychopathology in high‐risk professionals. The search identified 546 articles, 23 of which met eligibility criteria. Twenty interventions were prevention‐oriented, and three were treatment‐focused. Improvements in social support outcomes and PTSD symptoms were reported across studies, while evidence for other post‐trauma psychopathology was limited. For both preventive and therapeutic interventions, higher effectiveness was associated with more sessions, active skill training and sustained engagement with support sources. Peer support was specifically associated with effectiveness in preventive interventions, while partner support emerged as particularly beneficial in treatment settings. Moreover, treatment‐oriented interventions reported more substantial effect sizes compared to preventive interventions, but further research is needed to confirm this pattern. Results indicate that interventions should focus on training social support skills (e.g., support‐seeking behaviours) and promoting active and sustained engagement with available support sources to effectively prevent or treat PTSD symptoms. Future studies should explicitly evaluate social support as an active component of both preventive and therapeutic interventions to establish its role as a mechanism of change.

## Summary

- Interventions incorporating social support improve PTSD symptoms but are less effective for other post‐trauma psychopathology.
- Multi‐session interventions that include active engagement with support sources are associated with long‐term improvements in mental health and social support outcomes.
- Incorporating peer support into preventive interventions may enhance the effectiveness of these interventions.
- Active, group‐based social support skills training effectively improves mental health and social support outcomes.
- Therapeutic interventions, particularly those incorporating partner support, yield more substantial improvements for PTSD and social support outcomes than preventive interventions.

When Hank Schrader, a fictional law enforcement officer from the popular series *Breaking Bad*, is advised to see a psychiatrist following a traumatic incident at work, he responds with ‘Start going down that road, kiss your career goodbye’. This statement exemplifies some commonly held beliefs about seeking mental health treatment among high‐risk professionals, mainly the fear of being perceived as unfit for duty, concerns over confidentiality and anxieties about potential negative impacts on one's career (Haugen et al. 2017; Richards et al. 2021). High‐risk occupations include various professions, such as first responders (i.e., police officers, firefighters and paramedics), combat‐exposed military personnel and other professionals who are frequently exposed to potentially traumatic incidents at work (Pietrantoni and Prati 2008). These incidents are associated with an increased risk of developing various mental health problems, such as symptoms of post‐traumatic stress disorder (PTSD), depression, anxiety, substance abuse and suicidal ideation (Klimley et al. 2018; Syed et al. 2020). Given the nature of these professions, exposure to potentially traumatic events (PTEs) is often inevitable. However, the development of trauma‐related psychopathology may be mitigated through targeted prevention efforts. For such interventions to be effective, they must focus on specific, modifiable risk and protective factors (Jacka et al. 2013). Among these, social support consistently emerges as a key protective factor for mental health (e.g., Grover et al. 2024; Klimley et al. 2018).

The stress buffering hypothesis outlines the key role of social support for individuals at risk for trauma‐related psychopathology (Shallcross et al. 2016). According to this hypothesis, social support becomes particularly important during highly stressful or potentially traumatic events, as it can attenuate stress reactivity and thereby mitigate the risk of negative mental health outcomes (Cohen and Wills 1985).

## 1 Social Support and Mental Health Outcomes

Social support includes both structural aspects (i.e., network size) and functional (i.e., relationship quality) dimensions (Drageset 2021). Perceived support is a functional subdomain that reflects an individual's perception of the availability and suitability of support and is the most commonly measured facet of social support (Guilaran et al. 2018). Social support operates across multiple levels, ranging from close interpersonal relationships (e.g., partners) to family and community networks and even broader national and international structures (Sippel et al. 2015). This highlights that social support is a multidimensional construct, with each facet potentially exerting distinct influences on psychological outcomes (Guilaran et al. 2018).

Evidence from various high‐risk professions underscores the protective role of social support in mental health. In firefighters, lower perceived support from colleagues and supervisors is prospectively associated with higher PTSD symptomatology (Lommen 2024). Among police officers, a workplace culture that encourages emotional expression and higher perceived availability and satisfaction with support serves as a buffer against PTSD (Klimley et al. 2018). Among military personnel, perceived social support shows consistent cross‐sectional and longitudinal associations with probable PTSD and depression (Grover et al. 2024). In emergency nurses, higher perceived support from supervisors was associated with lower levels of fatigue, distress and somatic complaints, while support from colleagues was associated with reduced fatigue (Adriaenssens et al. 2012). Social support is also critical in exceptional circumstances marked by intense occupational stress, such as during the COVID‐19 pandemic. A study of healthcare workers during and after the first wave of the pandemic found some key differences among those who experienced short‐term or long‐term distress and those demonstrating resilience. Specifically, distressed workers reported a longer duration of occupational stress and perceived lower availability of organisational support (Rapisarda et al. 2023). The consistent associations between social support and reduced mental health problems highlight the preventive potential of supportive environments in high‐risk professions.

### 1.1 Social Support in Prevention and Therapeutic Contexts

Organisations frequently implement interventions to mitigate trauma‐related psychopathology following traumatic workplace incidents (Anderson et al. 2020). These prevention‐based approaches are generally perceived as useful and acceptable by public service personnel such as firefighters and police officers, in part because they are tailored to their organisational cultures (Corthésy‐Blondin et al. 2022). Moreover, a recent systematic review and meta‐analysis concluded that interventions designed to proactively mitigate the impact of traumatic workplace events (e.g., peer support and stress‐reduction programmes) can significantly reduce absenteeism and turnover in public safety and healthcare sectors, thereby reducing organisational costs (Azadehyaei et al. 2025).

Peer support programmes are the most commonly implemented preventive interventions in high‐risk professionals (Anderson et al. 2020). These programmes are led by trained peers who draw on their lived experience to help normalise post‐traumatic reactions, reduce stigma around seeking mental health support, and promote early symptom identification (Klimley et al. 2018). Peer support programmes support early prevention efforts by serving as a ‘first line of defense’ after a PTE (Rodriguez et al. 2024). Psychological debriefing interventions such as Critical Incident Stress Debriefing (CISD) are also widely used (Anderson et al. 2020). While these debriefing interventions can be delivered by various facilitators, such as therapists or social workers (Billings et al. 2023), the original debriefing model specifies the involvement of trained peer facilitators and a mental health professional (Mitchell 1983). Interventions that follow this model can be classified as peer support interventions.

Social support‐oriented prevention programmes effectively alleviate mental health symptoms, but might become ineffective once symptoms reach clinical levels, highlighting the need for appropriate specialised care (Guilaran et al. 2018). These therapeutic interventions can greatly benefit from integrating social support. In practice, this might involve engaging family, friends or partners to help sustain treatment engagement and adherence. In firefighters, for instance, these types of strategies have been shown to improve motivation to seek treatment and treatment adherence (Johnson et al. 2020).

### 1.2 Current Evidence and Research Gap

To date, systematic reviews evaluating both preventive and therapeutic interventions have generally found limited evidence supporting their effectiveness among high‐risk professionals. For example, a systematic review that included seven RCTs of interventions such as Trauma Risk Management (TRiM) and team‐based skills training for first responders found limited evidence for the effectiveness of these interventions on PTSD, anxiety or depression (Tan et al. 2022). Similarly, another systematic review exploring both prevention and treatment interventions for public safety personnel exposed to traumatic work incidents found that trauma‐focused treatments were generally associated with improvements in mental health symptoms, whereas the evidence for prevention programmes such as psychoeducation and resilience training was more limited (Corthésy‐Blondin et al. 2022). The most comprehensive systematic review to date evaluated various workplace psychosocial interventions (e.g., debriefing, TRiM, EMDR, CBT and Psychological First Aid) across multiple professional groups, including first responders and medical personnel. This review found an overall lack of effectiveness for preventing PTSD symptoms (Billings et al. 2023). While peer support programmes such as psychological debriefing and TRiM were covered in all aforementioned reviews, none of them explicitly acknowledge peer support or other forms of social support as mechanisms that may drive intervention effectiveness. This gap highlights the need for a broader examination into the role of social support in both the prevention and treatment of mental health problems among high‐risk professionals.

### 1.3 The Current Review

In light of this research gap, we conducted a scoping review to identify and describe studies that incorporate social support as a component in the prevention or treatment of post‐trauma psychopathology across high‐risk occupational groups. Following the scoping review criteria outlined by Munn et al. (2018), this review does not aim to quantify intervention effectiveness. Instead, the objective is to identify interventions that incorporate social support in various forms and highlight the aspects that influence their effectiveness in addressing post‐trauma psychopathology.

The current review adds to the existing literature in several respects. First, it offers a broad examination of social support as a component, which includes mode of delivery (e.g., peer‐delivered), specific intervention strategies (e.g., support‐seeking modules) and the enhancement of perceived social support as a targeted outcome. To identify these features, the review will focus on intervention studies with a range of study designs (i.e., quantitative, qualitative and mixed methods). Second, it expands the scope of mental health outcomes beyond PTSD, acknowledging the wider range of psychological challenges to which high‐risk professionals are vulnerable (Billings et al. 2023; Syed et al. 2020). Third, the review includes all high‐risk professionals, recognising that the impact and effectiveness of social support may vary across professional categories due to differences in factors such as organisational culture and structure (Guilaran et al. 2018).

## 2 Methods

This review followed the six‐step methodological framework of Arksey and O'Malley (2005) and adhered to the PRISMA‐ScR reporting guidelines (Tricco et al. 2018). The scoping review protocol and full search strategy were pre‐registered on the Open Science Framework (OSF; osf.io/nuf8g).

### 2.1 Search Strategy

The search strategy was drafted by the first and third authors and finalised in consultation with the last author. The search was conducted on 28 March 2025, using three academic databases: PsycInfo, Web of Science (WoS) Core Collection and Medline (through the WoS interface). The full search term is listed in Appendix S1.

### 2.2 Selection Process

The search yielded 546 results, which were uploaded to the programe Rayyan (Ouzzani et al. 2016) for screening. Three reviewers independently conducted the selection process, with each article being reviewed by at least two reviewers. After deduplication, 521 articles were screened, 473 of which were excluded based on title and abstract. A further 25 articles were excluded based on a full‐text review, leaving 23 articles to be included in this study. The screening was carried out in three distinct sets, each involving a different combination of reviewers. Due to the complexity of this process, a simple and readily interpretable descriptive measure of interrater agreement was selected. The interrater agreement was calculated by dividing the number of corresponding ratings by the total number of articles reviewed per set. The average of these three pair combinations was then used as a measure of interrater agreement, which was 96% for title and abstract screenings and 81% for the full‐text screenings. During both stages, discrepancies between reviewers were discussed and resolved in a consensus meeting.

### 2.3 Inclusion and Exclusion Criteria

In line with the objectives of this review, peer‐reviewed articles were included that investigated programmes or therapies with a social support component and were intended to prevent or treat post‐trauma psychopathology. These interventions were included if they focused on occupations that (potentially) exposed professionals to traumatic incidents. Studies that examined healthcare personnel during COVID‐19 were also included, given the significant occupational stressors during this period, such as risk of infection and death (Rapisarda et al. 2023). In addition, interventions aimed at fostering resilience were included, as these can be regarded as preventive in nature (Doody et al. 2021).

Articles were excluded if they did not report primary research, examined trauma unrelated to work in a high‐risk profession or did not involve an intervention. Studies were also excluded when they did not focus on post‐trauma psychopathology or were written in a language other than English or Dutch. In addition, articles were left out that were not available in full text during the screening stage. Grey literature was not included as previous research indicates that it requires a substantial time investment but yields little relevant evidence (Hartling et al. 2017).

### 2.4 Data Extraction

The data extraction form was developed through discussion between the reviewers, with the aim of systematically collecting relevant information from the selected articles. Extracted data included article details (authors, publication date and country), sample characteristics (sample size, age, gender, clinical status and type of high‐risk occupation), study design (objectives, design, control group and follow‐up), intervention or programme features (duration, type of social support, delivery mode and purpose: prevention/treatment), measures of psychopathology and social support and intervention effects (estimates, effect sizes, significance and moderators/mediators).

The included articles were divided into three sets, with data from each set extracted by one reviewer and cross‐checked by another. The obtained data were entered into a Microsoft Excel file.

After extraction was completed, data were organised across studies based on the intervention's primary purpose (i.e., prevention or treatment), and subsequently grouped by intervention type.

## 3 Results

### 3.1 Sources of Evidence

**FIGURE 1:**
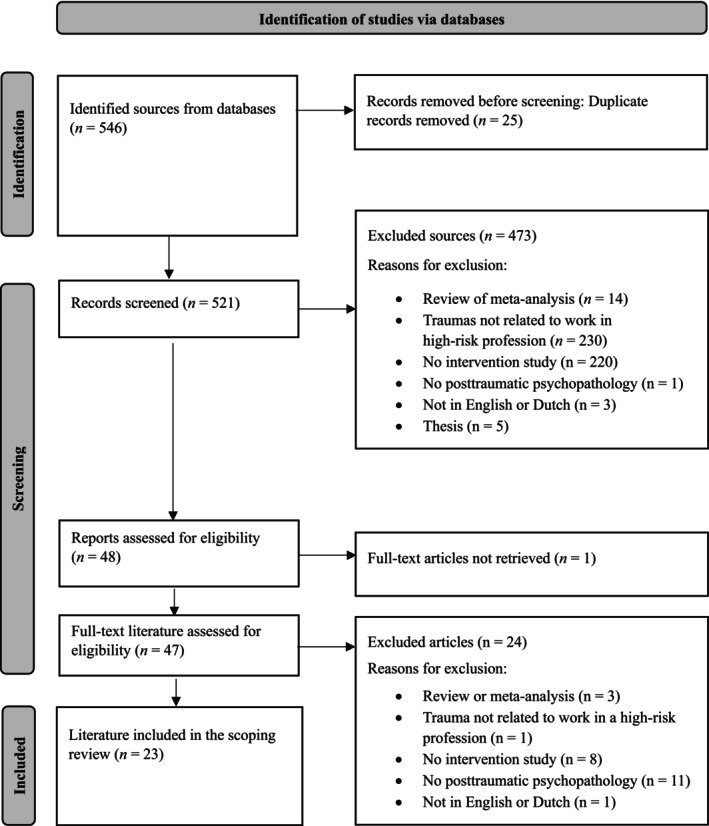
Flow chart of the study selection process.

The search yielded 546 results, of which 25 were duplicates. During the title and abstract screening stage, 473 articles were excluded, leaving 48 articles for full‐text review. One article could not be retrieved. Following the full‐text screening, 23 articles were included in the final review (see Figure 1).

### 3.2 Study Characteristics

Details about the characteristics of prevention‐oriented and treatment‐focused interventions, as well as key findings, can be found in Table 1. Of the included studies, 20 were categorised as prevention‐oriented and three as treatment studies, as they were conducted with clinical samples. Psychological debriefing was delivered in various forms, such as CISD, Critical Incident Stress Management (CISM), Post Critical Incident Seminar (PCIS) and Post‐Code Pause (*n* = 5). Three studies compared multiple interventions, most of which involved debriefing. Other preventive peer‐delivered interventions included TRiM (*n* = 5), Post‐Traumatic Intervention Team (PITT), Resilience and Coping for the Healthcare Community (RCHC) and Emotional Resilience Skills. Three interventions featured a social support module (i.e., I Was There Workshop, the Support Coach app, and Before Operational Stress). Treatment studies examined Cognitive‐Behavioural Conjoint Therapy (CBCT), Prolonged Exposure (PE) and a Veterans Transition Programme.

**TABLE 1:** Study characteristics and summary of main findings.

	Authors, year, country	Population (Sample size)	Intervention (duration/no. of sessions)	Social support component	Study design	Control group	Assessment time points	Mental health outcomes (Measures)	Social support (Measures)	Main findings
1	Greenberg et al. (2010) UK	Military (1235)	TRiM (15–22 months)	Peer‐delivered	RCT	Standard care	Baseline (T1) 12–18 months follow‐up (T2)	PTSD (PCL‐C) Psychological health (GHQ)	N/A	There was no difference between groups on mental health outcomes.
2	Hunt et al. (2013) UK	Police (640)	TRiM (N/A)	Peer‐delivered	Observational	No intervention	Baseline (T1) 1‐month follow‐up (T2)	Sickness absence (proxy for mental ill health) Risk of mental health disorders (TRiM risk checklist)	N/A	TRiM was associated with a reduction in sickness absence, especially in junior ranks. The TRiM group had a significantly lower risk of post‐incident psychopathology at follow‐up.
3	Jones et al. (2017) UK	Military (638)	TRiM (≥ 72 h after the traumatic even; 1 month later)	Peer‐delivered	Observational	Exposed non‐TRiM Non‐exposed non‐TRiM	6–12 weeks post‐deployment (T1) 1–2 year follow‐up (T2)	PTSD (PCL) Depression & anxiety (PHQ2 & GAD2) Alcohol misuse (AUDIT‐QF) Mental ill‐health caseness	Seeking formal and informal support	Compared to exposed non‐TRiM participants, exposed TRiM recipients were more likely to report possible PTSD symptoms at baseline (but not follow‐up), more likely to report persistent mental ill‐health caseness (e.g., possible anxiety, depression), and less likely to experience remission. At follow‐up, TRiM recipients were more likely to seek help from mental health services. There was no difference among groups in seeking mental health support from informal sources.
4	Frappell‐Cooke et al. (2010) UK	Military (180)	TRiM (≥ 72 h after the traumatic even; 1 month later)	Peer‐delivered	Observational	TRiM‐naive unit (in the initial stages of using TRiM)	4–6 weeks pre‐deployment (T1) Approx. 3 months into combat (T2) Within 1 week post‐deployment (T3)	PTSD (PCL‐C) General distress (GHQ‐12)	Perceived social support during tour of duty Unit social support	Pre‐deployment, the TRiM‐naive unit reported greater psychological distress (particularly among the never‐deployed), and higher trauma‐related stress (especially among the previously deployed). There were no significant post‐deployment differences for either outcome. During deployment, perceptions of unit support slightly increased for the TRiM‐experienced group, but both groups reported reduced unit support post‐deployment. Post‐deployment, perceived support during the tour did not differ significantly between groups.
5	Watson and Andrews (2018) UK	Police (859)	TRiM (N/A)	Peer‐delivered	Cross‐sectional	No intervention	N/A	PTSD (PCL‐C)	N/A	There was no difference in PTSD symptoms between the two groups.
6	Adler et al. (2008) US	Military (952)	CISD (N/A)	Peer‐delivered (+ mental health professionals)	RCT	Stress management class Survey only (no intervention)	Pre‐deployment recruitment (T1) Enrollment (T2) 3/4‐month follow‐up (T3) 8/9‐months follow‐up (T4)	PTSD (PCL‐C) Depression (CESD) Aggression (CTS) Alcohol problems (AUDIT)	Organisational support (POS)	Contrast analyses revealed small positive effects (*d* = 0.11) of CISD (vs SMC) on PTSD (between T1 and T3) and small effects (*d* = 0.10) on aggression (vs. SO) for soldiers with high PTE exposure (between T1 and T4). CISD group showed slightly higher post‐deployment AUDIT scores than other groups. Overall, soldiers perceived less organisational support over time. However, CISD was associated with higher organisational support for those with high PTEs, compared to no intervention.
7	Adler et al. (2009) US	Military (1060)	BD (26 sessions); SBT, (23 sessions); LBT (6 sessions)	Peer‐delivered	RCT (randomised by platoon)	Stress education	Baseline (T1) Post‐treatment (T2) 4‐month follow‐up (T3)	Distress (SUDS) PTSD (PCL) Depression (PHQ‐D) Sleep problems (NVS)	NVS	All Battlemind interventions (vs control) led to fewer PTSD symptoms, but only among those with highest combat exposure: *d* = 0.21 (BD); *d* = 0.14 (SBT); *d* = 0.16 (LBT). For depression, only BD was more effective than control for high‐exposure soldiers (*d* =. 26), while large‐group training reduced depression regardless of exposure level (*d* = 0.30). Sleep problems significantly improved for those with highest combat exposure: *d* = 0.25 (BD); *d* = 0.25 (SBT) *d* = 0.27 (LBT). Battlemind debriefing was seen as most effective for improving unit cohesion, as well as communication and felt understanding from team leaders. Small‐group training was most effective in supporting reintegration with family and friends.
8	Ruck et al. (2013) UK	Prison staff (91)	CISD (N/A)	Peer‐delivered (+ mental health professionals)	Observational	No intervention	Pre‐debriefing (T1) 1 month post‐intervention (T2)	PTSD (IESE) Anxiety (GAD) Depression (GAD)	N/A	The debriefed group showed significant improvement in PTS symptom scores between the two time points. No other effects were significant.
9	Price et al. (2022) CA	Firefighters and paramedics (215)	CISM (N/A)	Peer‐delivered	Quasi‐experimental	CISM unknown fidelity No CISM	N/A	AUD (AUDIT) GAD (GAD‐7) PD (PDSS‐SR) MDD (PHQ‐9) PTSD (PCL‐5) SAD (SIPS)	NVS (Peer support survey)	Participants in the high fidelity CISM condition were significantly less likely to screen positive for AUD and GAD than those with no CISM. For other mental health outcomes, the effects were comparable to the control condition. Of all the mental health outcomes measured, only PTSD was significantly associated with lower perceived peer support skills.
10	Carleton et al. (2020) CA	PSP (4020)	CISM, CISD, MHFA, Peer support training, R2MR (N/A)	Peer‐delivered	Cross‐sectional	Comparison between mental health training categories and a no training group	N/A	PTSD (PCL‐5) MDD (PHQ‐9) GAD (GAD‐7) SAD (SIPS) PD (PDSS‐SR) AUD (AUDIT)	NVS (Attitudes towards support)	Receiving any training was associated with lower odds of PTSD, MDD, GAD, and SAD compared to no training. After adjusting for demographics and other mental health training categories, few significant results remained. CISD was uniquely associated with decreased odds of screening positive for any mental health disorder. Spousal support was the most commonly endorsed early resource, while leadership support was most often endorsed as a last resort across all mental health categories. Participants with no training were less willing to access support from a peer or leadership.
11	Andrews et al. (2022) CA	Coast Guard personnel (412)	CISM, CISD, MHFA, Peer support training, R2MR (N/A)	Peer‐delivered	Cross‐sectional	Comparison between mental health training categories and a no training group	N/A	PTSD (PCL‐5) MDD (PHQ‐9) PD (PDSS‐SR) GAD (GAD‐7) SAD (SIPS) AUD (AUDIT)	NVS (Attitudes towards mental health support)	Having any mental health training (vs no training) was associated with a lower prevalence of all mental health disorders. Few significant results were unique to each mental health category. After adjusting for demographics, only peer support was linked to reduced odds of MDD. No significant results remained after adjusting for other mental health training categories. Across categories, participants preferred to turn to physicians first, then spouses, then friends, and least often to leadership or chaplains. Those without mental health training preferred to seek help from a spouse or friend instead of colleagues or leaders.
12	Copeland and Liska (2016) US	ESP (116)	Post‐Code Pause (10–15 s)	Peer‐delivered	Mixed methods	No control group	Baseline (T1) Mid‐programme (T2) Post‐programme (T3) 6‐month follow‐up (T4) 1‐year follow‐up (T5)	NVS	NVS	There were improvements from pre‐ to post‐implementation in various areas: perceived peer support (4%) and leader support (3%), time to regroup (18%), and ability to honour patients (25%), and a decrease in intrusions of traumatic event (21%). There was also an 11% increase in perceived pressure to return to work too quickly.
13	Korpela and Nordquist (2024) FI	ESP (15)	PCIS (3 days)	Peer‐delivered (+ mental health professionals)	Qualitative	No control group	6‐month follow‐up	Acute stress (N/A)	N/A	Participants reported greater openness to peer support, improved relationships and coping, and significant emotional relief following the intervention.
14	Fichera et al. (2015) IT	Bank employees (383)	Post‐robbery group support programme (N/A)	Organisational support	Mixed methods	No control group	Within 7–15 days of robbery (T1) 45 days post‐intervention (T2)	N/A	N/A	Most participants felt that the programme provided emotional support and mitigated post‐traumatic reactions. The opportunity to share experiences with peers was considered the most useful feature.
15	Guay et al. (2017) CA	Social service employees (222)	PTIT (N/A)	Peer‐delivered	Observational	No peer support, Standard organisational support	Baseline (T1) 2‐month follow‐up (T2) 6‐month follow‐up (T3) 1‐year follow‐up (T4)	ASD (ASDS) PTSD (PCL‐5) Depression (BDI) Anxiety (BAI)	JCQ (supervisor and co‐worker support subscales)	The peer support intervention showed no significant effect on any mental health outcomes compared to no peer support. Peer support and supervisor support did not predict any mental health outcomes.
16	Carleton et al. (2025) CA	PSP (119)	Emotional Resilience Skills Training (13 weeks)	Peer‐delivered	Observational	No control group	Pre‐training (T1) Post‐training (T2) 1‐year follow‐up (T3)	PTSD (PCL‐5) MDD (PHQ‐9) GAD (GAD‐7) SAD (SIPS) PD (PDSS‐SR) AUD (AUDIT)	N/A	There were significant changes in all mental health outcomes from pre‐ to post‐training and from pre‐ to follow‐up for PSP collectively (s). Firefighters and police showed significant improvements across outcomes (except SAD for firefighters and PD for both), with firefighters showing the largest overall effects. Paramedics showed improvements in MDD and GAD, while communicators showed reductions in PTSD and GAD.
17	Drebing et al. (2023) US	Veterans (40)	I Was There workshop (3 days)	Peer‐delivered	Observational	No control group	Baseline (T1) Post programme (T2) 1‐month follow‐up (T3) 4‐month follow‐up (T4)	PTSD (PCL‐5) MDD (PHQ‐8)	NVS (OQ post‐program perceptions of community interest)	A reduction in PTSD symptoms was observed from baseline to the 1‐month follow‐up. Most veterans reported increased willingness to share experiences with others, as week as seek support and connect with peers; most audience members reported increased interest and understanding of veterans' experiences.
18	Powell et al. (2022) US/PR	Healthcare personnel (476)	RCHC (2 sessions) RCHC+ (4 sessions)	Peer‐delivered (+ a mental health professional), Collective coping component	nRCT	Comparison between the two formats	Baseline (T1) 12‐week follow‐up (T2) 18‐week follow‐up (T3)	PTSD (IES‐R) GAD (BAI) Burnout (ProQOL) STS (ProQOL)	N/A	Both RCHC and RCHC+ significantly reduced PTSD symptoms at 12 and 18 weeks. Anxiety and STS symptoms improved in both groups, though only RCHC+ showed sustained improvement at week 18. Neither condition significantly reduced burnout.
19	Stelnicki et al. (2021) CA	PSP (136)	Before Operational Stress (12 months)	Communication, empathy and FD/FR modules Peer feedback in maintenance phase	Observational	No control group	Pre‐BOS (T1) 2 months post‐BOS active phase (T2) 1 month into maintenance phase (T3) 4 months into maintenance phase (T4) 7 months into maintenance phase (T5) 10 months into maintenance phase (T6)	PTSD (PCL‐5) Depression (DASS‐21) Anxiety (DASS‐21) Stress (DASS‐21) AUD (AUDIT) Resilience (BRS)	Perceived social support (SPS‐10)	The only significant effects were improvements in PTSD symptoms (*d* = 0.11) and social support, sustained through Time 4 (*d* = 0.17).
20	van der Meer et al. (2020) NL	Healthcare personnel (259)	Support Coach app (1 month)	‘Find support’ module	RCT	No intervention	Baseline (T1) Post‐condition (T2) 1 month follow‐up (T3)	PTSS (PCL‐5) Resilience (RES) Negative trauma‐related cognitions (PTCI)	Social support (SSL‐6)	The only significant effects were a decline in negative post‐trauma cognitions post‐intervention (*r* = 0.18) and at follow‐up (*r* = 0.20), and an increase in psychological resilience only at follow‐up (*r* = 0.15). 34% perceived the app as effective in increasing their comfort with seeking support.
21	Monson et al. (2024) US	Military and Veterans (32)	CBCT and PE (15 weekly sessions CBCT; 12 weekly sessions PE)	Partner support	RCT	Active control PE	Baseline (T1) Post‐treatment (T2) 3‐month follow‐up (T3) 6‐month follow‐up (T4)	PTSD (CAPS‐5); (PCL‐5)	SORTS	Large effects for PTSD in both conditions across time points (between *g* = 0.97 and *g* = 1.5 for PE and *g* = 1.1 and *g* = 1.7 for CBCT). CBCT was uniquely associated with improved partner‐rated PTSD symptoms (*g* = 0.1.9). It was also associated with improved partner accommodation, but not significantly different from PE.
22	Price et al. (2018) US	Military (123)	PE (8–12 weekly sessions)	Interaction between treatment and social support	Observational	No control group	Baseline (T1) 1 week post‐treatment (T2) 3‐month (T3) 6‐month follow‐up (T4)	PTSD (PCL‐M)—self‐report PTSD (CAPS)—clinician‐administered	Social support (MOS‐SSS)	There were improvements in CAPS, PCL, and MOS‐SSS scores during treatment only. Social support unidirectionally moderated PTSD changes during treatment.
23	Cox et al. (2019) CA	Veterans (218)	Veterans Transition Programme (10 days)	Solidifying interpersonal goals	Observational	No control group	1st day of programme (T1) 10th day (T2) 3‐month follow‐up (T3)	PTSD (PCL‐5)	Tangible, appraisal, belonging support (ISEL‐SF)	Reductions in PTSD from pre‐ to follow‐up (*d* = 0.1.1). All types of social support increased from pre‐ to follow‐up: *d* = 0.24 (tangible); *d* = 0.37 (appraisal); *d* = 0.35 (belonging), but none predicted changes in PTSD. However, higher pre‐programme PTSD levels attenuated changes in appraisal support.

*Note:* Effect sizes: *d* = Cohen's d, *g* = Hedge's g, *r* = Spearman rank correlations. Effect sizes were included only where explicitly reported in the original studies and the magnitude followed the classification provided by the authors.

Abbreviations: ASDS, Acute Stress Disorder Scale; AUDIT, Alcohol Use Disorders Identification Test; BAI, Beck Anxiety Inventory; BD, Battlemind Debriefing; BDI, Beck Depression Inventory; BRS, Brief Resilience Scale; CAPS‐5, Clinician‐Administered PTSD Scale for DSM‐5; CBCT, cognitive–behavioural conjoint therapy; CEQ, Community Experiences Questionnaire; CISD, Critical Incident Stress Debriefing; CISM, Critical incident stress management; CTS, Conflict Tactics Scales; DASS‐21, Depression, Anxiety and Stress Scale; GAD, Generalised Anxiety Disorder scale; GHQ, General Health Questionnaire; IESE, Impact of Event Scale; ISEL‐SF, Interpersonal Support Evaluation List—Short Form; LBT, Large Battlemind Training; MHFA, Mental Health First Aid; MOSS‐SSS, Medical Outcomes Study Social Support Survey; nRCT, non‐randomised cluster trial; NVS, non‐validated survey/self‐constructed questionnaire; PCL‐5, PTSD Checklist for DSM‐5; PCL‐C, PTSD Checklist—Civilian Version; PDSS‐SR, Panic Disorder Severity Scale; PE, Prolonged Exposure; PHQ, Patient Health Questionnaire; POS, Perceived Organisational Support; PTCI, Posttraumatic Cognitions Inventory; PTE, potentially traumatic events; PTIT, Post‐Traumatic Intervention Team; R2MR, Road to Mental Readiness; RCHC, Resilience and Coping for the Healthcare Community; RES, Resilience Evaluation Scale; SBT, Small Battlemind Training; SIPS, Social Interaction Phobia Scale; SSL, Social Support Scale; SORTS, Significant Others’ Responses to Trauma Scale; STS, Secondary Traumatic Stress; SUDS, Subjective Units of Distress Scale; TRiM, Trauma Risk Management.

These studies employed various designs, including observational (*n* = 10), RCTs (*n* = 5), cross‐sectional (*n* = 3), mixed‐methods (*n* = 2), qualitative (*n* = 1), quasi‐experimental (*n* = 1) and a non‐randomised cluster trial (*n* = 1). The populations assessed were primarily military and/or veterans (*n* = 9), public safety personnel (PSP; *n* = 9) and healthcare personnel (*n* = 2). Additional studies focused on other high‐risk professions, with one study each on prison staff, social service employees and bank employee victims of robbery.

Various psychopathology outcomes were assessed, with PTSD symptoms being most common (*n* = 18), frequently measured using the PCL scale. Other commonly assessed outcomes included alcohol problems (*n* = 7, measured with the AUDIT questionnaire), depression (*n* = 11) and anxiety (*n* = 9), assessed with various instruments (e.g., PHQ and GAD). Social support was evaluated in 15 studies, mostly using established instruments (e.g., SPS‐10 and POS) but also through non‐validated surveys, such as questions on informal support‐seeking or attitudes towards support.

### 3.3 Prevention‐Oriented Interventions

#### 3.3.1 Psychological Debriefing

Debriefing‐based interventions were typically peer‐delivered with support from a mental health professional. Two RCTs reported small positive effects of debriefing interventions compared to either an active condition or no intervention in soldiers with high occupational stress. These included improvements in PTSD and aggression (Adler et al. 2008), as well as PTSD, sleep symptoms and depression (Adler et al. 2009).

Another observational study found large effect sizes on PTSS symptoms for CISD versus no intervention among prison staff, although the short follow‐up (i.e., 1 month) and non‐experimental design limit the strength of these findings. When applied with high fidelity, CISM may be associated with unique benefits for alcohol problems and generalised anxiety, at least among firefighters and paramedics (Price et al. 2022). Two cross‐sectional studies with overlapping datasets reported similar psychopathology outcomes in PSP across various mental health training categories, including CISD and CISM (Andrews et al. 2022; Carleton et al. 2020). Overall, there was little to no evidence of effectiveness for outcomes such as generalised anxiety, social anxiety or panic disorder.

Interventions with multiple sessions, such as battlemind debriefing and small‐group battlemind training, emerged as particularly effective in enhancing perceived unit cohesion, communication with team leaders, and reintegration with family and friends (Adler et al. 2009). Moreover, individuals who received any form of mental health training (e.g., CISM, CISD or peer support training) were more likely to seek support from peers and leaders compared to those without such training (Andrews et al. 2022; Carleton et al. 2020). Qualitative findings among emergency personnel also suggest that debriefing enhances perceived social support, particularly peer and leadership support (Copeland and Liska 2016; Korpela and Nordquist 2024).

#### 3.3.2 Trauma Risk Management

TRiM is a peer‐delivered intervention that was mainly evaluated in military samples. Overall, there was little support for the effectiveness of TRiM on outcomes such as PTSD, depression, anxiety and alcohol use. However, one study found that TRiM was associated with reduced sickness absence and lower risk of psychopathology at 1‐month follow‐up in a police sample (Hunt et al. 2013). Two observational studies compared TRiM groups with either a non‐TRiM group (Frappell‐Cooke et al. 2010) or a group in the initial stages of using TRiM (Jones et al. 2017). Both studies found that TRiM recipients reported elevated PTSD symptoms at baseline but not at follow‐up, suggesting possible intervention benefits in military settings. Findings regarding social support were mixed, with one study reporting a decrease in unit support post‐deployment (Frappell‐Cooke et al. 2010), while another found improvements in support‐seeking (Jones et al. 2017).

#### 3.3.3 Other Peer‐Delivered Interventions

Three observational studies evaluated other peer‐delivered interventions and found mixed results. One study comparing various PSP groups found significant improvements in outcomes such as PTSD, depression and anxiety that were sustained at the 1‐year follow‐up, with firefighters showing the largest effect sizes (Carleton et al. 2025). Another study found reductions in PTSD symptoms and improvements in perceived and community support within 1 month of the intervention among veterans (Drebing et al. 2023). The one study with no significant effects was conducted among social service employees and was the only one to include a control group (Guay et al. 2017).

#### 3.3.4 Social Support as a Programme Component

Three studies evaluated interventions that explicitly included a social support component; two of these were conducted with healthcare worker samples. Two studies reported small effect sizes, reflecting sustained improvements in outcomes such as PTSD and secondary traumatic stress. One peer‐delivered intervention included a module on collective coping skills (Powell et al. 2022), while the other featured a maintenance phase with 10 monthly meetings, during which peers provided feedback on skill implementation (Stelnicki et al. 2021). The latter also found increased perceived social support. Among the three studies, only the self‐guided app intervention included a randomised control group and showed no significant effects on PTSD symptoms or perceived social support (van der Meer et al. 2020).

### 3.4 Treatment Studies

Only three treatment studies were identified, all involving military or veteran samples diagnosed with PTSD. One RCT compared the effects of CBCT and an active control (PE), both involving partners in the intervention (Monson et al. 2024). The study found large effect sizes corresponding to sustained improvements in self‐rated and clinician‐rated PTSD in both conditions, with partner‐rated PTSD and partner accommodation being uniquely associated with CBCT. The other two studies, which lacked control groups, examined the social causation versus social erosion models. The social causation model posits that strong social support protects individuals from developing PTSD, while the social erosion model proposes that PTSD erodes social support resources (Wang et al. 2021). Price et al. (2018) found that improvements in social support moderated PTSD changes during a PE intervention, providing support for the social causation model. Cox et al. (2019) found large effect sizes for PTSD and small effect sizes for social support after a 10‐day Veterans Transition programme. Consistent with the social erosion model, pre‐programme PTSD attenuated changes in social support, while social support did not predict PTSD symptom changes.

## 4 Discussion

The present review aimed to identify and describe social support interventions for preventing or treating post‐trauma psychopathology in high‐risk professionals. A total of 23 studies were included, comprising 20 prevention‐oriented programmes and three treatment‐focused programmes.

Most preventive interventions were peer support programmes that were either exclusively peer‐delivered (e.g., TRiM and PITT) or co‐delivered with a mental health professional (e.g., CISM and CISD). Three preventive interventions explicitly incorporated social support components. Treatment studies examined partner support and tested the social causation and social erosion models. In terms of social support outcomes, perceived social support was the most frequently measured, consistent with prior research in high‐risk professionals (Guilaran et al. 2018). A few studies measured attitudes towards receiving support and support‐seeking behaviours, which are essential for the quality and accessibility of social support (Feeney and Collins 2015).

Overall, findings provide support for the effectiveness of these interventions in improving social support outcomes and reducing PTSD symptoms, although evidence for other post‐trauma psychopathology outcomes remains limited. The social support features associated with intervention effectiveness and the evidence for incorporating social support strategies into interventions are discussed below.

### 4.1 Preventive Interventions

Prevention‐oriented studies typically assessed multiple psychopathology outcomes, but effects were mainly found for PTSD. Most interventions included, such as TRiM and debriefing‐based interventions, are selective prevention programmes that focus primarily on mitigating PTSD symptoms (Billings et al. 2023). By contrast, universal prevention programmes typically incorporate various elements (e.g., psychoeducation and coping skills acquisition), making them better suited for addressing a broader range of mental health concerns (Corthésy‐Blondin et al. 2022).

Overall, preventive interventions yielded small effects, consistent with broader research on social support, which typically reports small to medium effect sizes (Guilaran et al. 2018). Nonetheless, programmes with more sessions and richer content, such as Battlemind debriefing and training, Emotional Resilience Skills Training and Before Operational Stress, were associated with improvements in mental health that were sustained for a longer time. This supports prior meta‐analytic findings indicating that the number of sessions is a significant moderator of intervention effectiveness, particularly for PTSD (Alshahrani et al. 2022).

#### 4.1.1 Peer Support and Mental Health

Most prevention‐oriented studies reported significant PTSD effects, suggesting that peer support may be associated with intervention effectiveness. By contrast, the most comprehensive review of post‐incident psychosocial interventions in high‐risk professionals to date reported more mixed evidence (see Billings et al. 2023). One explanation is that our review's focus on social support led to the inclusion of primarily peer‐delivered interventions, while Billings et al. (2023) examined similar interventions (e.g., psychological debriefing, TRiM) delivered by a wider range of providers, including chaplains and social workers. However, none of the included interventions explicitly evaluated peer support as a component. This is consistent with the broader literature, as mechanisms of action are rarely examined in peer support interventions (Lloyd‐Evans et al. 2014). Debriefing interventions, such as CISD and CISM, are particularly challenging to assess because social support effects cannot be distinguished from clinician effects. Moreover, even though peer facilitation is a key feature of these interventions (Mitchell 1983), only one study examined adherence to the peer‐support model, which was uniquely associated with effects on alcohol use disorder (AUD) and generalised anxiety disorder (GAD).

While results suggest that peer support may enhance intervention effectiveness, the current data do not allow for definitive conclusions, as this scoping review was designed to map existing evidence rather than quantify component‐specific effects. Nevertheless, this presents a promising direction for future research.

#### 4.1.2 Social Support as an Intervention Component

Three studies explicitly incorporated social support as a programme component, mainly in the form of social support skills and peer support. One study compared a brief and extended version of RCHC, a peer‐delivered intervention that included a module on collective coping skills (Powell et al. 2022). The extended version was associated with mental health effects that were maintained over a longer period of time. Another intervention combined interpersonal skills modules with a 10‐month maintenance phase of monthly peer‐feedback meetings and found sustained improvements in PTSD and social support (Stelnicki et al. 2021). The third intervention was an app featuring a ‘find support’ module and found reductions in negative post‐trauma cognitions but no improvements in PTSD or social support (van der Meer et al. 2020). These findings suggest that active, group‐based skills training is more effective than non‐interactive support modules. Importantly, even though social support was conceptualised as a mechanism of action in these studies, it was not tested independently as a component.

#### 4.1.3 Social Support Outcomes

Even though social support components were not explicitly assessed, many studies measured social support as an outcome, indicating it is often a treatment goal and providing preliminary evidence for incorporating it into preventive programmes. TRiM was linked to greater support‐seeking, which is a key aim of this intervention (Greenberg et al. 2010). However, it was also associated with a decrease in perceived unit support over time, suggesting the programme may be too brief to sustain social support. The benefit of longer interventions is highlighted by a study comparing Battlemind debriefing (26 sessions) with small‐group (23 sessions) and large‐group (6 sessions) Battlemind training, as only the first two resulted in enhanced social support outcomes (Adler et al. 2009). For other debriefing interventions, both quantitative and qualitative studies suggest modest improvements in organisational, peer and leadership support, as well as attitudes towards seeking support and related behaviours. Another peer support intervention, the 3‐day workshop *I Was There*, which encouraged active, goal‐focused collaboration, yielded improved social support 1 month post‐intervention.

Findings on attitudes and support‐seeking behaviours underscore the importance of incorporating strategies that foster engagement with support networks into interventions (Sippel et al. 2015). Evidence on perceived support further suggests that multisession interventions and active engagement with support sources are particularly effective for sustaining support.

### 4.2 Treatment Studies

In contrast to prevention studies, treatment studies showed large effects for PTSD and small to medium effects on social support. This aligns with the findings of Corthésy‐Blondin et al. (2022), who reported consistent effects of trauma‐focused treatments on PTSD and limited evidence for selective preventive interventions such as CISM and TRiM. These interventions are usually brief and implemented as a first aid solution after a critical incident (Mitchell 1983; Greenberg et al. 2010). As such, they can be considered ‘low intensity’ interventions and generally yield small effects that require larger sample sizes to detect (Matthay et al. 2021). Moreover, preventive post‐incidence interventions are often applied universally rather than targeting individuals at heightened risk (Tan et al. 2022). This could partially explain the magnitude of effects, given that interventions designed primarily for a subset of a population yield smaller effect sizes when applied to the entire population (Matthay et al. 2021). By contrast, the identified clinical studies applied evidence‐based interventions, such as PE and CBCT, to individuals with probable PTSD or a PTSD diagnosis. This targeted approach is more likely to produce greater symptomatic improvement (Matthay et al. 2021). These interventions also consist of multiple sessions. This offers more ‘room’ for symptomatic improvement, as individuals gain deeper insight into their experience and develop more effective coping strategies over multiple sessions (Thompson‐Hollands et al. 2023).

This converges with preventive intervention studies, where more sessions and richer content (e.g., in Battlemind debriefing and Before Operational Stress) were associated with sustained improvements in mental health and social support outcomes. This has important clinical implications, as brief programmes are more cost‐effective in the short term, but longer programmes yield greater clinical benefits and quality‐of‐life improvements over time and may reduce future mental health‐related costs (Azadehyaei et al. 2025).

Partner support emerged as a promising intervention target given that the study reporting the largest effects on PTSD and social support incorporated partner support into treatment (see Monson et al. 2024). This aligns with previous findings that the active involvement of significant others in treatment is effective in reducing PTSD symptoms (Meuleman et al. 2023). Additionally, treatment studies provided preliminary support for both the social causation and social erosion models. Price et al. (2018) found that social support moderated changes in PTSD symptoms (social causation), whereas Cox et al. (2019) found that PTSD reduced social support over time (social erosion). These findings mirror broader literature, with reviews reporting evidence for both processes (Wang et al. 2021). This might be explained by an initial buffering effect of social support on PTSD in the aftermath of a traumatic event, followed by a gradual decline in support over time, which ultimately reverses the association (Sippel et al. 2015).

### 4.3 Limitations of Existing Evidence

Although we did not formally assess methodological quality, we identified one pattern that warrants further discussion. Studies using a cross‐sectional or pre–post design more often reported significant effects than controlled studies. This is a critical observation, as PTSD rates tend to decline naturally over time (Cukor et al. 2011). Without a control group, it becomes difficult to distinguish treatment effects from natural recovery, expectancy effects or setting influences (Cuijpers et al. 2017). Among the RCTs, which provide the highest level of evidence, the findings are mixed. One study reported strong effects on PTSD (Monson et al. 2024), two found no effects (Greenberg et al. 2010; van der Meer et al. 2020), and two reported effects only under specific conditions, such as high trauma exposure or particular time contrasts (Adler et al. 2008; Adler et al. 2009). This discrepancy between controlled and uncontrolled studies limits the strength of evidence for social support interventions.

Most of the included studies focused on peer support. We identified only one study about partner support and one about organisational support despite our broad search for various types of social support. This narrow focus is noteworthy, as different forms of social support may have distinct effects on PTSD trajectories (Wang et al. 2021). Future research should explore a wider range of support types to better understand their respective roles in support erosion and intervention outcomes.

Another limitation is that only three treatment studies were identified. These studies generally reported larger effect sizes than preventive interventions, which prompts further investigation into the robustness of this trend across additional studies. Moreover, given evidence that support can erode over time (Wang et al. 2021) and that people with clinical PTSD report lower social support (Grover et al. 2024), further research on incorporating social support into psychotherapeutic interventions is warranted.

Lastly, most of the studies were conducted in high‐income countries, specifically the United Kingdom, United States and Canada, which limits the generalisability of the findings. Prior research shows that first responders in middle‐ and low‐income countries have higher PTSD rates than those in high‐income countries (Arena et al. 2025). Cross‐country differences may be especially pronounced for certain high‐risk occupations, such as military personnel whose deployment experiences can vary widely across countries (Grover et al. 2024). In addition, national policies differ in how they support high‐risk professionals exposed to PTEs—for example, the United Kingdom offers TRiM as a standard intervention (Stileman and Jones 2023). Although it is well known that cultural differences affect the provision and receipt of social support (Sippel et al. 2015), most research on social support and PTSD has been conducted in Western countries (Wang et al. 2021). As a result, little is known about how cross‐cultural differences shape the effectiveness of social support interventions for PTSD in non‐Western settings. Future research should therefore investigate social support interventions for high‐risk professionals across a wider range of cultural contexts.

### 4.4 Strengths and Limitations of the Current Review

Given the limited research on social support in intervention settings, we chose a scoping review methodology to investigate this topic. The broad search strategy facilitated the identification of various social support mechanisms (e.g., peer support and social support intervention modules). Social support components were not explicitly examined as mechanisms of change, meaning evidence regarding mental health improvements cannot be directly attributed to social support. Moreover, we only selected articles in English and Dutch to ensure interrater reliability. This may have led to the omission of relevant papers published in other languages. This is especially pertinent given the scarcity of knowledge on social support and mental health in non‐Western countries (Wang et al. 2021).

## 5 Conclusion

The results of this scoping review provide tentative evidence for the effectiveness of social support interventions, particularly for reducing PTSD symptoms in high‐risk professionals. Preventive peer‐delivered interventions with multiple sessions, as well as treatments that actively incorporate social support, appear most effective for sustaining improvements in PTSD and perceived social support. Incorporating a social support component into treatment may be especially promising, as it was associated with greater improvements, though the available evidence remains limited. Most studies did not explicitly assess social support as an intervention component, making it difficult to determine whether observed effects can be attributed to social support processes. Additionally, differential effects between controlled and uncontrolled studies, limited evidence for different types of social support and overreliance on data from Western countries limit the conclusions that can be drawn about the effectiveness of these interventions in high‐risk professionals.

Despite these limitations, the review highlights the promising role of social support in trauma‐related interventions. Future research should aim to evaluate social support as an active intervention component to establish its role as an effective mechanism of change.

The Appendix S1 outlines the search terms and search fields applied in PsycInfo and Web of Science (Web of Science Core Collection and MEDLINE).

## Conflicts of Interest

The authors declare no conflicts of interest.

## Supporting information


**Appendix S1:** Search strategy for PsycInfo and Web of Science.

## Data Availability

The study protocol is available on Open Science Framework (osf.io/nuf8g). Data supporting findings are available in the article and Supporting Information. Access to the data extraction table is available upon request to the corresponding author.
