# Supplementary material for: Social Support Interventions and Post‐Trauma Psychopathology in High‐Risk Professionals: A Scoping Review
Source: Clin Psychol Psychother. 2026 Apr 15;33(2):e70274. doi: 10.1002/cpp.70274 (PMC13080587; doi:10.1002/cpp.70274)
Supplement: Supplementary file 1 — Appendix S1: Search strategy for PsycInfo and Web of Science. [file CPP-33-e70274-s001.docx]

**Appendix S1**

**Search strategy for PsycInfo and Web of Science**

Search field in Web of Science: Topic (title, abstract, keywords)

Search field in PsycInfo: Title or abstract (XB)

Social support intervention* or Social support therapy or Social support program* or

Psychosocial intervention or Peer support or Companionate support or Workplace support or

Coworker support or Formal social support or Institutional support or Familial support or

Relational support or Organisational support or organizational support or Supervisor support

AND

High-risk or High risk or Firefight* or Fire fighters or First responders or Police or Paramedics

or Public safety personnel or PSP or Law enforcement or Military or soldiers or Emergency

dispatchers or Emergency service

AND

PTSS or PTSD or Posttraumatic or Post-traumatic or Post-trauma or Trauma-related

AND

Traumatic events or PTE or Critical incident
